# Association between physical activity and online sexual objectification experience: The mediating role of body-image depression

**DOI:** 10.3389/fpsyg.2022.1049588

**Published:** 2023-01-10

**Authors:** Xiang-Yu Du, Lin Wang, Yi-Fan Zuo, Qing Wu, You-Ling Qian, Rui Ma

**Affiliations:** ^1^School of Physical Education, Wuhan University of Technology, Wuhan, China; ^2^School of Physical Education, Hubei Minzu University, Enshi City, China; ^3^Postdoctoral Research Station in Public Administration, School of Physical Education, Zhengzhou University, Zhengzhou, China

**Keywords:** mental health, online sexual objectification experience, body-image depression, physical activity, female college students, mediating role

## Abstract

**Objective:**

With the popularization and development of online media technology, more and more women are paying attention to their body image and physical behavior. The purpose of this study was to investigate the effect of online sexual objectification experience on the physical activity of female college students and verify the mediating role of body-image depression between them.

**Methods:**

A cross-sectional convenient sample of 882 female college students from four universities in Hubei Province completed an online survey, and the Online Sexual Objectification Experience Scale (OSOES), the Body-Image Depression Questionnaire, and the Physical Activity Rating Scale (PARS) were used to collect the data. The mediating effect of the association between online sexual objectification experience and physical activity, was examined using the process procedure in SPSS and the bootstrap method.

**Results:**

Online sexual objectification experience was significantly positively correlated with physical activity (*r* = 0.420, *p* < 0.01). Body-image depression was significantly negatively correlated with online sexual objectification experience and physical activity (*r* = −0.484, *p* < 0.01; *r* = −0.569, *p* < 0.01). Online sexual objectification experience can affect physical activity directly (β = 6.49, *p* < 0.001, effect value 44.97%) and also indirectly through body-image depression (β = 7.95, *p* < 0.001, effect value 55.03%); there were significant differences between major and education-level categories in body-image depression and physical activity.

**Conclusion:**

Both online sexual objectification experience and body-image depression can promote physical activity among female college students, and body-image depression has a mediating effect between online sexual objectification experience and physical activity.

## Introduction

With the popularization and development of online media technology, social media is used to widely promote photos or short videos containing slimming, beauty, and body appearance content. Female college students, who are in an important period of physiological development and are more sensitive to their physical appearance, are prone to excessive self-objectification, such as posting selfies or being exposed to others’ beautified photos and giving feedback (likes and comments) anytime and anywhere (de Valle et al., 2021). Previous research has found that self-objectification is more prevalent in young women and can trigger a range of physical and mental health problems, such as inducing external manifestations of disordered eating behaviors, excessive body monitoring, and body shame (Fredrickson et al., 1998; Zhang et al., 2021). However, the experience of online sexual objectification is a common form of self-objectification experience, specifically an experience of extensive attention to the appearance on social media that encompasses both media and interpersonal approaches to objectification and can significantly predict others’ sense of objectification (Luo, 2017). A growing body of research suggests that social media provides opportunities to objectify women because it often displays idealized and inauthentic images of glamor, which can easily trigger the internalization of thinness ideals and social comparisons (Saiphoo and Vahedi, 2019). A previous meta-analysis found that women have a richer experience of objectification than men and that younger women are more likely to be troubled by online sexual objectification and body image problems than older women (Mingoia et al., 2017). The general population is more likely to compare themselves to people at similar levels, rather than models and celebrities, and a recent study found that body image comparisons between peers were more helpful in reinforcing female college students’ online sexual objectification experience (Tiggemann et al., 2018) and that such comparisons tended to provide more valid evaluative information (Festinger, 1954). In addition, we found that with the increased use of appearance-focused social media (e.g., Tik Tok and Facebook), the effect on women’s online sexual objectification and body image problems was stronger (Huang et al., 2021). Therefore, exploring online sexual objectification is important for promoting the mental health of female college students.

Physical activity has now been identified as a “meaningful” activity that enhances body self-concept, body self-esteem, and body satisfaction (Richman and Shaffer, 2000; Babic et al., 2014). The World Health Organization (WHO) defines physical activity as any physical activity produced by skeletal muscles that requires energy expenditure higher than that at the metabolic level at rest (Guthold et al., 2020). Studies have shown that regular participation in physical activity can be beneficial for moderate weight loss and shaping in exercising populations (Tucker and Mortell, 1993), and can contribute to the prevention of mental health problems such as anxiety, depression, and eating disorders (Dale et al., 2019; Mathisen et al., 2021). A review of the literature found that self-objectification promotes physical activity, health quality, and body confidence in women (Ginis et al., 2012). Although it has been well documented that self-objectification positively predicts female physical activity, the relationship between online sexual objectification through social media and female college students’ physical activity has not been established. Thus, this article explores the relationship between physical activity and online sexual objectification experience for female college students.

However, research on online sexual objectification experience and physical activity has mainly focused on whether objectification positively affects physical activity, and there is a paucity of research on how it alters the effects of physical activity and through what paths it leads individuals to increase their physical activity. In particular, the frequency of body image comparison (mutual evaluation) on social media, users uploading messages, the sharing of photos, and other inter-individual comparisons have led to young women being overly concerned with their body image, having a strong desire to improve their bodies, and having negative emotional experiences. Many studies have confirmed that young women have higher negative body image awareness and face stronger body image pressure than men, and female college students, as an important representative group of young women, are characterized by rapid physiological development and a high level of body image concern (Smolak, 2009) and are prone to symptoms of body-image depression such as appearance anxiety. Body image is also known as body imagery, and body-image depression is a kind of abnormal body image psychology caused by the deviation of an individual’s perception of the real body image and ideal body image (Liang et al., 2017). Recent studies show that body-image depression is growing more common in the world, with a detection rate of body-image depression among female college students in China of 28.7% (Zhang and Liu, 2019) and an incidence of body image disturbance in Korea of 51.8% (Hyun et al., 2014). This is especially prominent in Western countries, where 80% of female college students report dissatisfaction with their bodies (Myers and Crowther, 2009). If an individual has severe body-image depression, it can affect their social life, interpersonal relationships, studies, and mood, and long-term body-image depression can also lead to a series of mental health problems such as low self-esteem (Gao et al., 2006), eating disorders (Thomas et al., 2010; Stice et al., 2011), and depression (Rierdan and Koff, 1997; Jones and Griffiths, 2015). Fredrickson’s objectification theory suggests that women who objectify themselves are prone to body shame, appearance anxiety, etc., and are motivated to improve their body image and have a good body experience, thus actively engaging in physical activity (Fredrickson and Roberts, 1997). Therefore, the body-image depression of female college students should also be considered. Based on these findings, this study aims to clarify the relationship between online sexual objectification experience, physical activity, and body-image depression among female college students, as well as their internal mechanisms, in order to provide a reference for promoting psychological well-being interventions among female college students.

## Literature review and research hypothesis

### Direct effects of online sexual objectification and physical activity

The self-objectification theory suggests that the experience of objectification can cause women to habitually apply a third-party perspective to themselves, engage in frequent self-monitoring of their appearance, be more sensitive to body-related information, and be more likely to feel dissatisfied with their body image, which can put women at risk of psychological disorders related to appearance anxiety, body dissatisfaction, and a reduced experience of flow states (Fredrickson and Roberts, 1997). A review of the literature found that participation in two physical activities or longer periods of physical activity predicted lower levels of self-objectification in women compared with women who did not participate in physical activity (Slater and Tiggemann, 2011, 2012). A recent cross-sectional study explained the mechanism of self-objectification by suggesting that participation in physical activity moderates the association between the internalization of the thinness ideal and self-objectification. This may be due to the importance of internal focus during physical activity, which allows for a better respect and appreciation of one’s body, thus reducing the frequency of self-objectification and negative emotional experiences, and helping women to regain confidence in their appearance (Jankauskiene and Baceviciene, 2022). This finding provides evidence to support that self-objectifying women engage in more physical activity. In addition, Parsons and Betz (2001) found that women with different levels of objectification tended to choose different exercise programs to alleviate their self-objectification, and that this also had a significant influence on the environment and effects of physical activity (Dimas et al., 2021). Accordingly, we propose research hypothesis H1: online sexual objectification experience positively influence physical activity among female college students.

### Indirect effects of online sexual objectification and physical activity

Based on objectification and social comparison theories, objectified women continuously monitor their bodies and habitually compare their bodies to popular societal aesthetic standards (upward comparison), which makes them prone to body-image depression and dissatisfaction with their bodies (Zhao et al., 2021). Previous evidence suggests that social media environments with a richer online sexual objectification experience compared with traditional media are associated with higher levels of body-image depression among female college students (Cohen and Blaszczynski, 2015). This may be because social media creates ample conditions for online sexual objectification experience to occur, and women are more worried about when and how their bodies will be exposed to social media; this uncertainty dramatically increases rates of body-image depression in women (Mingoia et al., 2017). One study found that online sexual objectification is potentially harmful to individuals and is a risk factor for body-image depression (Fardouly and Vartanian, 2016). In addition, female college students with high levels of self-objectification not only reinforce self-objectification but also objectify others, forming a “cycle of objectification” mechanism that increases female college students’ body-image depression (Strelan and Hargreaves, 2005). We propose research hypothesis H2: online sexual objectification experience can positively predict female college students’ body-image depression.

In addition, many studies have confirmed that better body image is associated with physical activity; for example, Kirkcaldy et al. (2002) found that regular physical activity was associated with higher body satisfaction, possibly because physical activity can improve one’s body shape or appearance to some extent, enhancing individual self-efficacy and confidence in the control of one’s appearance, thus alleviating body-image depression (Abbott and Barber, 2011). It was found that athletes who regularly participated in physical activity had a more positive body image than non-athletes (Soulliard et al., 2019). The results of two other meta-analyses consistently concluded that body image was positively associated with physical activity and confirmed a slight to moderate effect on body image improvement (Campbell and Hausenblas, 2009; Bassett-Gunter et al., 2017). Indeed, in addition to altering one’s appearance, physical activity may also have a potential effect on body image perception, although this relationship may be more complex due to its association with health promotion and physical strength (Li et al., 2016). A previous review found that physical activity may have a positive effect on body image perception (Sabiston et al., 2019). A recent study similarly confirmed this finding, particularly that moderate and vigorous physical activity was strongly associated with body image problems in young women and adolescents (Miranda et al., 2021). Thus, for individuals with body-image depression, participation in physical activity can be an effective way to manage weight and improve body satisfaction (Bray et al., 2016). However, it is worth noting that physical activity is a “double-edged sword” for improving body image and that the excessive or prolonged misuse of physical activity can also have adverse physical and psychological health consequences (Lichtenstein et al., 2017). Physical consequences mainly manifest in long-term risks such as pain, physical deformities, and repetitive injuries (Tod et al., 2016). Psychological impairment is usually manifested as a range of negative emotional and physical symptoms, such as anxiety, depression, irritability, and mood changes that can induce psychological problems such as social disorders and eating disorders (Weinstein et al., 2015). The close association with eating disorders, in particular, is a common feature, with nearly 80% of patients with eating disorders exhibiting excessive physical activity (Bewell-Weiss and Carter, 2010; Bratland-Sanda et al., 2010). Accordingly, we propose research hypothesis H3: body-image depression is an intermediate variable between online sexual objectification experience and physical activity.

**FIGURE 1:**
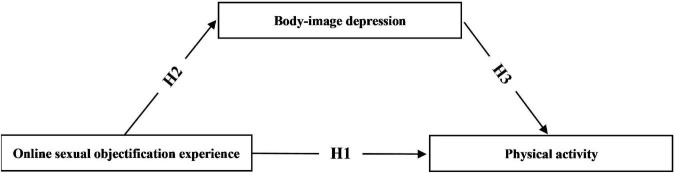
Research theoretical model.

As can be seen, there are a number of domestic and international studies focusing on the relationship between online sexual objectification experience and physical activity among female college students, and some literature has also explored the interaction between body-image depression, body dissatisfaction, and physical activity; however, there is little in-depth exploration of the predictive role of the three, especially using body-image depression as a mediating variable to verify the effect of online sexual objectification experience on physical activity. This field of research is still in its infancy in China, and systematic studies focusing on this topic are lacking. To sum up the above analysis, based on objectification theory, social comparison theory, and the triple-factor model of socio-cultural influence on individual body image proposed by Thompson et al. (1999), this study constructs a theoretical relationship between online sexual objectification experience, body-image depression, and physical activity among female college students, and integrates hypotheses H1, H2, and H3 to form a research structure (Figure 1). In addition, it was found in previous studies that major, education, place of birth, and being an only child may have different degrees of influence on the three research variables, so they were included in this study as covariates in the model to compare the differences in online sexual objectification experience, body-image depression, and physical activity scores. Given that college and university is an important time for the physical and mental health development of college students, and considering the prevalence of social media use among female college students, it is necessary to examine the online sexual objectification experience of female college students. Based on the existing theories and literature studies, this study analyses the correlation between the three factors and tries to explain the mechanism at the structural level, aiming to provide theoretical support and guidance for female college students to enhance their well-being, and provide a reference for relevant departments to make decisions.

## Research methods

### Participants

A total of 882 female college students from four universities in Hubei Province were selected by convenient sampling method. In order to ensure the quality of the test, the test was conducted in class under the coordination of the head teacher and teachers. The subjects were given instructions before distributing the questionnaire, and all the subjects participated voluntarily after understanding the purpose of the study. At first, 18 female college students said they did not intend to take the test. After the end of the test time, 24 female students did not take the test at all. According to the questionnaire recovery standard, they answered all the questions of the questionnaire within the prescribed time as required, and 858 questionnaires were collected on site. According to the principle of obviously wrong answers, random filling or incomplete, a total of 28 invalid questionnaires were excluded and 830 valid questionnaires were retained, with an efficiency rate of 96.7%, according to the principle of obvious misfiring, omission, or random filling of answers. The age range of the subjects was between 17 and 28 years old, with an average age of 21.17 ± 2.03 years; there are 394 undergraduates and 436 postgraduates, 190 in science and technology categories, 481 in arts categories, and 159 in arts and sports categories. The inclusion criteria of the participants in this study were healthy female college students who passed the entrance physical examination, consented, and volunteered to participate. The exclusion criteria were that they could not complete the questionnaire within the deadline and unwillingness to accept the survey. A few points need to be clarified: (1) All female university students participated in the test voluntarily, and the questionnaire was answered online and collected on the spot, with a filling time of 4 min. (2) Before the test, training was conducted for the test group composed of 10 psychology graduate students. (3) In order to avoid the disadvantages of self-reporting, the test was scored in a positive and negative way and anonymously. (4) All procedures were in accordance with the Declaration of Helsinki. (5) The study was authorized by the local Ministry of Education Institutional Review Board (102772021RT072), which is responsible for evaluating studies conducted at schools.

### Research tools and procedures

#### Online sexual objectification experience scale

The online sexual objectification experience scale developed by Luo (2017). was used to investigate female college students, which has a total of 6 questions and uses 5-point Likert scoring, using 5 levels, with a score of 1–5, respectively; the higher the score, the richer the individual’s online sexual objectification experience. In this study, Cronbach’s alpha coefficient of the scale was 0.883, and the index met the psychometric requirements.

#### Body-image depression questionnaire

The body-image depression questionnaire developed by Gao et al. (2005). was used to investigate body-image depression of female college students in four dimensions: body-shape depression, gender depression, sexual-organ depression, and appearance depression. The questionnaire consists of 25 items, including 8 items on body-shape depression, 4 items each on gender depression and sexual-organ depression, and 9 items on appearance depression, which has been widely validated in the female university student population (Zhang and Liu, 2019; Zhao et al., 2021). And the questionnaire was scored on a 3-point scale, i.e., 1 point for conformity, 2 points for non-controversial, and 3 points for non-conformity. The lower the score, the higher the level of body-image depression; conversely, the higher the score, the lower the level of body-image depression. A score of <2 for a single dimension indicates the presence of such worries and ≥2 indicates the absence of such worries (Luo et al., 2005). The Cronbach’s alpha scores ranged from 0.816 to 0.91 for each factor and 0.93 for the whole scale, demonstrating sufficient internal consistency.

#### Physical activity rating scale

The physical activity of female university students was measured using the Physical Activity Rating Scale developed by Hashimoto (1990), which was localized to take into account our national conditions. There are three questions in the scale to examine the amount of physical activity in terms of intensity, time, and frequency of physical activity, and each dimension is scored on a 5-point Likert scale. Exercise volume = intensity × time × frequency; exercise intensity, and exercise frequency, from 1 to 5 levels, scored 1–5 points; and exercise time from 1 to 5 levels, scored 0–4 points. The lowest score was 0 points, the highest score was 100 points, the higher the score indicating a greater amount of physical activity and the higher the level. The Cronbach’s alpha coefficient of PARS was 0.846, and the index met the psychometric requirements.

### Statistical analysis

After eliminating the unqualified questionnaires, the questionnaire numbers were entered into SPSS 25.0. An independent sample *t*-test was used to compare the differences in indicators of education-level categories, place of birth, and whether they were only children; one-way ANOVA was used to compare the differences in indicators between students of different majors; Pearson correlation analysis was used to explore the correlations between online sexual objectification experience, body-image depression, and physical activity among female college students; Harman one-factor test and validation factor analysis were used to test for common method bias. The procedure developed by Hayes was used to select model 4, test the mediating effect between online sexual objectification experience and physical activity of body-image depression, and adopt the bias-corrected bootstrap method (5,000 repetitions of sampling) to estimate 95% confidence intervals (CI) to further validate the mediating effect in order to achieve the study needs.

## Results

### Demographic results

A total of 830 female college students were included in this study, with online sexual objectification experience scores of 2.12 ± 0.55, body-image depression scores of 2.25 ± 0.34, and physical activity scores of 20.80 ± 18.91. The highest physical activity scores were (25.08 ± 20.68) for arts and sports students, and higher for arts (20.42 ± 18.89) than for science and technology (18.17 ± 16.83).

### Common method deviation control and inspection

Harman’s one-factor method was used to test for common method bias, and all question items about online sexual objectification experience, body-image depression, and physical activity were subjected to unrotated exploratory factor analysis; a total of six common factors with eigenvalues greater than 1 were extracted, and the maximum factor explained 34.954% of the total variance, which was less than 40% of the judgment criterion (Podsakoff et al., 2003). In addition, the validation factor analysis revealed that the one-factor model fit was poor (X^2^/df = 11.156, CFI = 0.637, TLI = 0.637, IFI = 0.638, RMSEA = 0.111), further indicating that there was no serious common method bias in this study and that statistical requirements had been met.

### Demographic variance analysis

From the *t*-test and one-way ANOVA results (Table 1), the online sexual objectification experience, body-image depression (including body-shape depression and appearance depression), and physical activity of female college students with different majors were significantly different, but gender depression and sexual-organ depression were not significantly different. Online sexual objectification experience, body-image depression, and physical activity were higher in arts and sports categories than in arts categories, and were higher in arts categories than in science and technology categories. There were significant differences in body-image depression (including body-shape depression and appearance depression) between female college students with different education levels categories, undergraduate students scored more highly than graduate students; however, there were no significant differences between these groups in online sexual objectification experience and physical activity. There were no significant differences in online sexual objectification experience, body-image depression, and physical activity between different birth sources or whether or not respondents were only children.

**TABLE 1:** Demographic characteristics of the sample.

Variables		*n*	Statistics	OSOE	BID	Body-shape depression	Gender depression	Sexual-organ depression	Appearance depression	PA
M	STC	190		2.05 (0.51)	2.30 (0.35)	2.27 (0.41)	2.28 (0.48)	2.27 (0.47)	2.35 (0.44)	18.17 (16.83)
	AC	481		2.09 (0.55)	2.26 (0.34)	2.21 (0.43)	2.26 (0.47)	2.31 (0.44)	2.30 (0.42)	20.42 (18.89)
	ASC	159		2.29 (0.56)	2.16 (0.33)	2.06 (0.38)	2.25 (0.43)	2.27 (0.43)	2.15 (0.44)	25.08 (20.68)
			*F*	10.322	8.765	12.571	0.113	0.786	10.476	6.064
			*p*	**	**	**	0.893	0.456	**	**
ELC	Undergraduates	394		2.09 (0.55)	2.28 (0.34)	2.23 (0.41)	2.26 (0.45)	2.28 (0.45)	2.33 (0.43)	21.46 (19.20)
	Graduates	436		2.15 (0.55)	2.23 (0.35)	2.17 (0.43)	2.27 (0.47)	2.30 (0.44)	2.23 (0.43)	20.19 (18.65)
			*t*	−1.353	2.138	2.250	−0.369	−0.536	3.306	0.966
			*p*	0.177	*	*	0.712	0.592	***	0.335
POB	Rural	427		2.16 (0.55)	2.26 (0.35)	2.20 (0.41)	2.27 (0.47)	2.29 (0.44)	2.29 (0.44)	19.68 (17.86)
	City	403		2.08 (0.55)	2.25 (0.34)	2.21 (0.42)	2.26 (0.46)	2.29 (0.45)	2.27 (0.43)	21.99 (19.92)
			*t*	1.870	0.249	0.023	0.200	0.084	0.421	−1.759
			*p*	0.062	0.803	0.832	0.842	0.933	0.674	0.079
OC	Yes	384		2.09 (0.56)	2.26 (0.35)	2.21 (0.43)	2.27 (0.46)	2.28 (0.46)	2.29 (0.43)	20.23 (18.99)
	No	446		2.15 (0.54)	2.24 (0.34)	2.19 (0.41)	2.25 (0.47)	2.29 (0.43)	2.27 (0.44)	21.28 (18.85)
			*t*	−1.391	0.951	0.744	0.628	−0.281	0.465	−0.800
			*p*	0.164	0.342	0.457	0.530	0.778	0.642	0.424

OSOE, online sexual objectification experience; BID, body-image depression; PA, physical activity; M, major; ELC, education-level categories; POB, place of birth; OC, only children; STC, science and technology categories; AC, arts categories; ASC, arts and sports categories. *P < 0.05; **p < 0.01; ***p < 0.001.

### Correlation analysis

Pearson’s correlation analysis of online sexual objectification experience, body-image depression, and physical activity, shown in Table 2, revealed that online sexual objectification experience was negatively correlated with body-image depression (*r* = −0.484, *P* < 0.01), body-image depression was negatively correlated with physical activity (*r* = −0.569, *P* < 0.01), and online sexual objectification experience was positively correlated with physical activity (*r* = 0.420, *P* < 0.01).

**TABLE 2:** Descriptive statistics and correlation analysis of all the variables.

Variables	Mean	SD	1	2	3
(1) Online sexual objectification experience	2.12	0.55	1		
(2) Body-image depression	2.25	0.34	−0.484**	1	
(3) Physical activity	20.80	18.91	0.420**	−0.569**	1

**P < 0.01.

### Body-image depression mediating-effect test

A multivariate hierarchical regression analysis using Process, an SPSS macro program developed by Hayes (2013), was used to test for mediating effects. After controlling for major and education level categories, regression analysis revealed that online sexual objectification experience significantly positively predicted physical activity (β = 14.229, *p* < 0.001) and significantly positively predicted body-image depression (β = −0.293, *p* < 0.001) giving support for H1 and H2. When both online sexual objectification experience and body-image depression predicted physical activity among female college students, the positive predictive effect of online sexual objectification experience on physical activity was still significant (β = 6.448, *P* < 0.001). Therefore, body-image depression played a partial mediating role between online sexual objectification experience and physical activity, as shown in Table 3.

**TABLE 3:** Regression analysis of the mediation model.

Result variables	Predictive variables	*R* ^2^	Δ*R*^2^	*F*	β	*t*
PA	Major	0.183	0.179	46.145***	–2.036	–1.702
	STC					
	AC				1.468	0.997
	ASC				3.443	1.850
	OSOE				14.229	12.986***
BID	Major	0.241	0.238	65.658***	–0.035	–1.661
	STC					
	AC				–0.027	–1.056
	ASC				–0.074	−2.273**
	OSOE				–0.293	–15.322***
PA	Major	0.358	0.354	92.049***	–2.956	−2.783**
	STC					
	AC				0.748	0.572
	ASC				1.482	0.895
	OSOE				6.448	5.855***
	BID				–26.541	–15.015***

OSOE, online sexual objectification experience; BID, body-image depression; PA, physical activity; STC, science and technology categories; AC, arts categories; ASC, arts and sports categories. *P < 0.05; **p < 0.01; ***p < 0.001.

The mediation effect was further tested using the bootstrap method with 5,000 random samples; if the confidence interval does not include 0, it indicates a significant mediation effect. The test results showed that the confidence interval of the mediating effect of body-image depression was [6.610, 9.380], excluding 0, indicating that body-image depression mediated the effect of online sexual objectification experience on physical activity of female college students. The total effect of online sexual objectification experience on physical activity was 14.44, of which the direct effect was 6.49 (accounting for 44.97% of the total effect), and the indirect effect through body-image depression was 7.95 (accounting for 55.03% of the total effect), this supports H3 in this study (Table 4).

**TABLE 4:** Bootstrap analyses of the mediation effect.

Effect	Effect value	95% confidence interval		Effect of the amount
		**Lower limit**	**Upper limit**	
Total effect	14.44	12.312	16.566	
Direct effect	6.49	4.335	8.651	44.97%
Intermediary effect	7.95	6.610	9.380	55.03%

## Discussion

The findings of this study showed that there was a significant correlation between online sexual objectification experience, body-image depression, and physical activity among female college students. The results proved that the richer the online sexual objectification experience of female college students, the more serious body-image depression and the more active the participation in physical activity. Further analysis revealed that the differences between online sexual objectification experience, body-image depression, including body-shape depression and appearance depression, and physical activity were significant between different majors. The differences between education level categories in body-image depression, body-shape depression, and appearance depression were significant, and the differences in online sexual objectification experience and in physical activity were not significant. The online sexual objectification experience and physical activity scores of arts and sports categories are higher than those of arts categories, and the scores of arts categories are higher than those of science and technology categories. This may be due to the characteristics of the majors, as art students have a strong aesthetic awareness, are more sensitive to their own body image, and often look at the bodies of others and themselves from an objectification perspective, resulting in a richer objectification experience compared to the other two categories of body-image comparison frequency; physical education students have formed a good awareness of physical activity and developed physical activity habits, and their attitudes and behaviors are both significantly higher than those of other majors (Moreno et al., 2014).

Hypothesis one of this study was verified. This study found that a certain level of online sexual objectification can positively predict physical activity. A study based on American women also confirmed this result, showing that self-objectification is an important predictor of physical activity in both young and older women (Rose, 2008). Self-objectified individuals have higher expectations for improving their appearance through physical activity, which leads women to choose physical activity to improve body satisfaction when comparing them to ideal beauty. Individuals with different levels of self-objectification also differ in the type of physical activity, motivation, and choice of environment for physical activity (Harper and Tiggemann, 2008). For example, individuals with high levels of self-objectification are more likely to choose appearance-oriented physical activity and also prefer fitness centers to sites outside fitness centers in terms of environment choice (Prichard and Tiggemann, 2008).

Hypothesis two of this study was verified. According to the study, the higher the level of online sexual objectification, the more serious the body-image depression, which is consistent with previous studies (Grippo and Hill, 2008). Social media is more popular than traditional media among female college students (Bair et al., 2012). Users in social media are both creators and receivers, and can post content as well as comment and agree with their companions (Prieler and Choi, 2014), this greatly enriches the online sexual objectification experience, and combined with the over-promotion of idealized female bodies inherent in the social media environment, creates fertile conditions for body-image depression among female college students.

Correlation and regression analyses showed a negative correlation between body-image depression and physical activity, that is, body-image depression promoted physical activity participation, indicating that when female college students have body-image depression, they may show more physical activity due to stimulation, hoping to build their body shape and improve their body image through active participation in physical activity. This is consistent with the results of the cross-sectional study of Gaspar et al. (2011). Numerous studies have shown that moderate physical activity can improve an individual’s body image, and most people participate in physical activity for the purpose of changing and maintaining their body shape and obtaining an attractive body shape (Furnham and Greaves, 1994). The study also found that individuals’ self-body image cognition is also one of the important factors affecting physical activity, which will not only affect physical activity behavior but also affect the emotion and attitude during physical activity (Hausenblas and Fallon, 2006). Physical activity can reduce the experience of objectification and alleviate body-image depression. First, because it is the most convenient and effective means of promoting the physical and mental health of college students. Physiological analysis suggests that weight loss and shape change is influenced by a negative energy balance. Adherence to exercise can directly promote metabolism, stimulating sugar and fat consumption, and thus reduce body weight. At the same time with diet control, take the form of dynamic and static combination effect is better (Jakicic and Davis, 2011). It is worth noting that exercise to improve body shape is a long-term physiological adaptation process, to achieve gradual and consistent progress, participants should choose the appropriate exercise program from their own situation; this should involve reasonable degrees of intensity, time, and frequency, preferably under the guidance of professional sports instructors in order to develop a scientific exercise prescription. Second, active participation in physical activity can effectively enhance self-efficacy and self-esteem levels and promote the development of human socialization (Ouyang et al., 2019). Physical activity can strengthen body perception and movement perception, and good proprioception not only helps college students better recognize their self-image, so reducing cognitive bias, but also allows them to fully immerse themselves in the fun of sports, thus enhancing individual self-confidence. Previous studies have found that individuals with different objectification levels tend to choose different types of sports, probably because individuals with high self-objectification are more likely to be driven by body-image comparisons, tend to be associated with more negative body-image outcomes, and are more involved in appearance-oriented sports; individuals with low self-objectification are more likely to be involved in health-oriented sports (Prichard and Tiggemann, 2008). Third, self-objectifying individuals believe that by being physically active they may achieve socially promoted standards of beauty, enhance friendships among peers, family, and other interacting groups, and reduce the risk of interpersonal objectification occurring. However, there is also an alternative argument that self-objectifying individuals reduce physical activity in order to avoid showing their “imperfect” bodies as much as possible (Leary, 1992). It remains to be studied which of the two opposing effects is stronger and which is weaker.

Hypothesis three of this study was tested. The findings suggest that online sexual objectification experience influence physical activity by affecting body-image depression. Online sexual objectification experience was a predictor of body-image depression (Bassett-Gunter et al., 2017), higher levels of online sexual objectification were associated with more severe body-image depression, and individuals with body-image depression may achieve their desired body image through physical activity (Campbell and Hausenblas, 2009).

The survey shows that the online sexual objectification experience score of female graduate students is higher than that of undergraduate students, but the level of body-image depression is lower than that of undergraduate students, which indicates that graduate students have more mature coping mechanisms when facing body-image depression. There may be several reasons for this phenomenon: First, compared with undergraduates, graduate students bear a greater psychological burden; the heavy academic and employment pressures directly lead to shorter exposure to social media. Research has found that reduced social media use time predicts lower body-image depression (Marengo et al., 2018). Therefore, the risk of body-image depression for graduate students is reduced. Second, the wide variety of sexualized images that flood social media is a significant contributor to body dissatisfaction among adolescent girls, and these images have a more negative impact on how undergraduate students in early and mid-adolescence view their bodies compared to the graduate student population in late adolescence (Tiggemann and Lynch, 2001). Third, graduate students are older and have richer experiences, have a deeper understanding of their body image, and have been able to internalize a more stable body image of themselves. So, they can respond in a positive way to the sexualized images in social media and reduce the frequency of appearance comparisons to reduce body-image depression (Tatangelo and Ricciardelli, 2017).

### Research limitations and prospects

The contribution of this study is mainly reflected in two aspects. Firstly, it reveals a clear and systematic structure that links online sexual objectification experience, body-image depression, and physical activity, and explores the mediating role of online sexual objectification experience and physical activity of body-image depression. Secondly, this study included an older group of graduate students than the previous group of college students. However, whether the current findings apply to women of other ages needs further research.

This study has the following limitations. Firstly, only a cross-sectional study was used and a follow-up survey is lacking, so the causal relationship could not be verified. It is hoped that follow-up research methods can be used in the future to further support the results of this study. Secondly, the sample of this study consisted of female college students, and future survey respondents should include diverse groups of young women including athletes, etc. The study found that female athletes have lower body satisfaction than non-athletes (Varnes et al., 2013). People are more concerned about female athletes’ body image and temperament rather than athletic performance (Fink, 2015), and female athletes are often promoted as attractive and sexy in the media environment, leading female athletes to over-surveil their body image and be more susceptible to body-image depression (Bissell, 2004). In addition, the data sources are all self-reported, and the subjects may be forced by social and cultural pressures to report better results than the actual results. Finally, although women experience objectification more than men, the impact of objectification on men cannot be ignored, and studies have found that men’s levels of objectification may be increasing in today’s media environment (Farquhar and Wasylkiw, 2007; Hatton and Trautner, 2011). Therefore, this study also needs to expand the sample to males in order to provide a theoretical basis for the relevant departments to scientifically formulate countermeasures, and promote the physical and mental health development of all college students. The physical activity measurement method of this study needs to be improved. A physical activity recorder should be used in the future to provide precise and real-time monitoring of physical activity data. Although further research is needed, existing studies have confirmed that self-objectification and body-image depression are important factors affecting the physical and mental health of contemporary college students. Future health studies should pay attention to this topic, and reduce the negative effects of self-objectification and body-image depression from the aspects of effective physical activity and improving the cognition of self-body image, so as to improve the mental health level of college students.

## Conclusion

This study found that major and educational level categories had significant effects on female college students’ online sexual objectification experience, body-image depression (body-shape depression, appearance depression) and physical activity. Online sexual objectification experience and body-image depression can positively predict the physical activity of female college students, and body-image depression has a significant mediating effect between online sexual objectification experience and physical activity.

## Data availability statement

The original contributions presented in this study are included in this article/supplementary material, further inquiries can be directed to the corresponding authors.

## Ethics statement

The studies involving human participants were reviewed and approved by the local Ministry of Education Institutional Review Board (102772021RT072). The patients/participants provided their written informed consent to participate in this study.

## Author contributions

X-YD, LW, and RM contributed to the design of the questionnaire. QW and Y-FZ organized and analyzed the database. X-YD, LW, and Y-LQ contributed to writing the manuscript. All authors contributed to the article and approved the submitted version.
